# Tunable Broadband Solar Energy Absorber Based on Monolayer Transition Metal Dichalcogenides Materials Using Au Nanocubes

**DOI:** 10.3390/nano10020257

**Published:** 2020-02-01

**Authors:** Jiakun Li, Zeqiang Chen, Hua Yang, Zao Yi, Xifang Chen, Weitang Yao, Tao Duan, Pinghui Wu, Gongfa Li, Yougen Yi

**Affiliations:** 1 Joint Laboratory for Extreme Conditions Matter Properties, Southwest University of Science and Technology, Mianyang 621010, China; lijiakun1999@yeah.net (J.L.); wtyao@ustc.edu.cn (W.Y.); myyz1984@swust.edu.cn (T.D.); 2 Research Center for Photonic Technology, Fujian Key Laboratory for Advanced Micro-nano Photonics Technology and Devices & Key Laboratory of Information Functional Material for Fujian Higher Education, Quanzhou Normal University, Fujian 362000, China; czqchem@qztc.edu.cn; 3 State Key Laboratory of Advanced Processing and Recycling of Non-ferrous Metals, Lanzhou University of Technology, Lanzhou 730050, China; hyang@lut.cn; 4 Key Laboratory of Metallurgical Equipment and Control Technology of Ministry of Education, Wuhan University of Science and Technology, Wuhan 430081, China; ligongfa@wust.edu.cn; 5 College of Physics and Electronics, Central South University, Changsha 410083, China; yougenyi@csu.edu.cn

**Keywords:** solar energy absorber, monolayer MoS_2_, local surface plasmon resonances, Au nanocubes, transition-metal dichalcogenides

## Abstract

In order to significantly enhance the absorption capability of solar energy absorbers in the visible wavelength region, a novel monolayer molybdenum disulfide (MoS_2_)-based nanostructure was proposed. Local surface plasmon resonances (LSPRs) supported by Au nanocubes (NCs) can improve the absorption of monolayer MoS_2_. A theoretical simulation by a finite-difference time-domain method (FDTD) shows that the absorptions of proposed MoS_2_-based absorbers are above 94.0% and 99.7% at the resonant wavelengths of 422 and 545 nm, respectively. In addition, the optical properties of the proposed nanostructure can be tuned by the geometric parameters of the periodic Au nanocubes array, distributed Bragg mirror (DBR) and polarization angle of the incident light, which are of great pragmatic significance for improving the absorption efficiency and selectivity of monolayer MoS_2_. The absorber is also able to withstand a wide range of incident angles, showing polarization-independence. Similar design ideas can also be implemented to other transition-metal dichalcogenides (TMDCs) to strengthen the interaction between light and MoS_2_. This nanostructure is relatively simple to implement and has a potentially important application value in the development of high-efficiency solar energy absorbers and other optoelectronic devices.

## 1. Introduction

As important energy collection devices, solar energy absorbers have attracted more and more attention in recent years [1,2,3]. For an ideal absorber, it is necessary to have a high light absorption and many other characteristics, such as polarization-dependence [4] and tunability [5]. However, problems arise as associated with limitations in low-temperature tolerance, low light absorption efficiency, and materials [6,7,8]. Therefore, we need to propose a new type of broadband absorber based on materials with excellent physical properties to solve these existing problems.

Over the past few years, 2D materials, such as graphene and transition-metal dichalcogenides (TMDCs), have become the leading materials for numerous applications in modulators, optical detectors, metamaterial absorbers, and emitters due to their extraordinary electrical and optical characteristics, and also their ability to enhance light absorption with high temperature stability [9,10,11,12,13,14,15,16,17,18,19,20,21,22,23,24,25]. Researchers have a great interest in metamaterial absorbers due to their wide range of applications in energy harvesting, sensing, and photocatalysis [26,27,28,29,30,31,32,33,34,35]. Monolayer TMDCs are regarded as a kind of semiconductor material and have a direct bandgap at the *K* points of the Brillouin zone [36], which means only energy will change in the transition process while the momentum remains unchanged. This process only needs the participation of photons and does not require additional phonons, which makes it much easier to generate and has a higher quantum efficiency. Compared with pure graphene, TMDCs have been considered as more preferable materials for applications, because pure graphene’s band-gap is zero [37] and its electrical conduction is only stimulated by electronic heat, which greatly limits its application in many fields. TMDCs have a tunable bandgap ranging from 1 to 2 eV [38], which provide opportunities for manufacturing photoelectron devices. Moreover, molybdenum disulfide (MoS_2_) has been considered as one of the most promising materials for realizing functional photonic devices due to its high current cut-off ratios and tunable optoelectronic characteristics [39,40]. Monolayer MoS_2_ shows excellent properties including direct band-gap and tunability, all of which are critical to optoelectronic devices manufacturing. However, due to its ultra-thin thickness, monolayer MoS_2_ also faces an intractable challenge in weak light absorption, which would hinder its practical application. We know that the average absorption rate of monolayer MoS_2_ is only 10% in the wavelength range from 450 to 800 nm [41]. Therefore, it is urgent to propose a useful method to improve the light absorption of monolayer MoS_2_.

In the last decade, an increasing number of strategies have been proposed for light absorption enhancement of monolayer MoS_2_. Based on the critical coupling mechanism, Xiao et al. [37] added an air chamber into a polymethyl methacrylate (PMMA)-SiO_2_-Ag three-dimensional structure, which greatly enhanced the absorption of monolayer MoS_2_. Hua et al. [42] enhanced Tamm Plasmon (TP) modes by inserting monolayer MoS_2_ into a grid composed of titanium dioxide and silicon dioxide, and then enhanced its absorption to 96%. Janisch et al. [43] raised the absorption of MoS_2_ to approximately 70% at 450 nm by using a nanocavity composed of alumina nano-layer spacers. Luo et al. [44] designed a perfect absorber based on metal-insulator-metal (MIM) metamaterial. The two absorption peaks of monolayer MoS_2_ increased to 57% and 80.5%.

Additionally, a useful method of enhancing and remotely controlling the optical response of materials depends on the use of local surface plasmon resonances (LSPRs) maintained by metal nanoparticles (NPs) [45]. The LSPRs wavelength of silver and gold nanoparticles can be effectively adjusted by changing the size, shape, and surrounding medium [46,47]. Liu et al. [48] proposed embedded nanostructures of noble metal NPs. Theoretical analysis and experimental results show that the coupling effect of LSPRs on an electromagnetic field and ferromagnetic (FM) material will be greatly changed due to the position change of nanoparticles. Mehrvar et al. [49] enhanced Raman scattering by chemical deposition, using the combination of LSPRs for silver nanoparticles, longitudinal standing wave resonance of the silver layer, and interaction between particles in the gap region. Sobhani et al. [50] used Au-Shell nanoparticles with a surface coverage of less than 1% to enhance the photocurrent and photoluminescence effects of molybdenum disulfide. In this work, we choose representative Au NPs-Au nanocubes (NCs) for simulation. Although light absorption was promoted by the introduction of Au NCs, it is still insufficient to apply to solar energy absorbers and other optoelectronic devices. An effective strategy is urgently needed to tackle this problem.

In this paper, we proposed a new method to enhance the absorption of monolayer MoS_2_ by using distributed Bragg mirror (DBR) as the substrate and combining with Au NCs periodic array nanostructures. DBR can effectively reduce light transmission and improve its reabsorption. Au NCs will facilitate us to adjust the optical properties of the nanostructure. Thus, a new type of high-efficiency broadband solar energy absorber working in the visible band was obtained. Additionally, through our theoretical calculation, other TMDC materials besides MoS_2_, such as WS_2_, MoSe_2_, and WSe_2_, can also be used in this nanostructure. These results indicate that the plasmon-enhanced nanostructures have wide applicability in the efficient solar energy absorption of two-dimensional materials.

## 2. Structure and Theory

**Figure 1:**
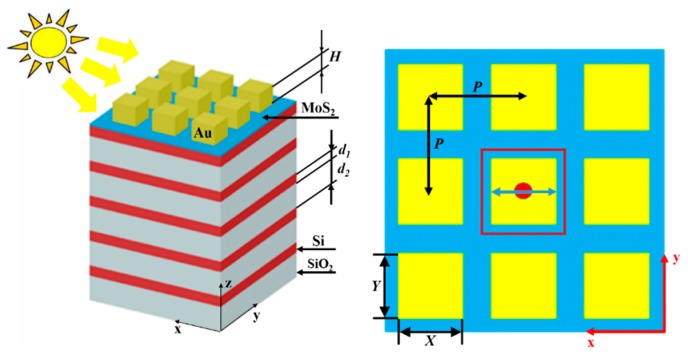
A schematic of the MoS_2_-based nanostructure. *X*, *Y*, and *H* represent the length, width, and height of the nanocubes, respectively. *d_1_* and *d_2_* are the thickness of Si and SiO_2_, respectively. *P* represents the period of the Au nanocubes (NCs) array.

Figure 1 shows the nanostructure we proposed. The periodic Au NCs array, the DBR composed of SiO_2_ and Si layers, and the monolayer MoS_2_ between the periodic Au NCs array and the DBR constitute this nanostructure. In this work, the dielectric properties of MoS_2_ were measured by Zhang et al. [51]. The Au NCs array is characterized by the period *P_x_* and *P_y_* in the *x* and *y* directions, respectively. The dielectric functions of SiO_2_ and Si are derived from Palik [52]. The thickness of the monolayer SiO_2_ layer is 90 nm and that of the Si layer is 38 nm. The incident optical source from air to the periodic Au NCs array is set for plane waves. The thickness of monolayer MoS_2_ is 0.65 nm. The dielectric function of Au is derived from Palik [52]. Finite-difference time-domain (FDTD) simulation is used to calculate the light absorption of the proposed MoS_2_-based nanostructure (Lumerical Solutions, Inc., Vancouver, YVR, Canada). Periodic boundary conditions are set around the unit cell, and the top and bottom boundaries of the cell are perfectly matched layer-absorbing boundary conditions. The formula for calculating absorption is *A* = 1 – *T* − *R* [53,54,55,56,57,58]. *T* represents transmission and *R* represents reflection.

## 3. Results and Discussions

**Figure 2:**
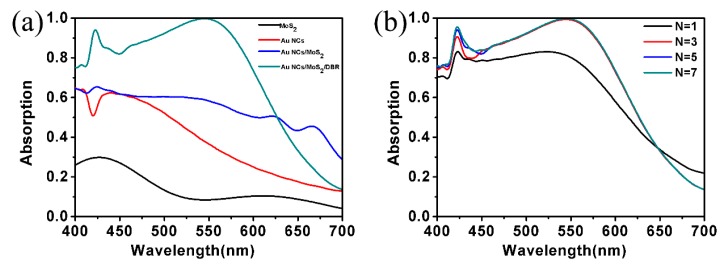
(**a**) Light absorption rate for Au NCs array, Au NCs/MoS_2_, Au NCs/MoS_2_/DBR, and monolayer MoS_2_. (**b**) Light absorption rate for different period number *N*. DBR: distributed Bragg mirror.

In this paper, the geometric parameters of the structure are set as follows unless it is explicitly pointed out that the parameters have changed: *X* = *Y* = *H* = 70 nm, *N* = 5, *d*_1_ = 38 nm, *d*_2_ = 90 nm, and *P* = 100 nm. The simulated absorption spectra results of the monolayer MoS_2_, a periodic Au NCs array, a periodic Au NCs array on monolayer MoS_2_ and the proposed MoS_2_-based nanostructure under transverse magnetic (TM)-polarized light are shown in Figure 2a. In the range of 400–700 nm visible light wavelength, the average absorptivity is about 0.1. The absorption at the peak of ~426 nm is about 0.29. Although the absorption coefficient of monolayer MoS_2_ is large, which can be considered as a transparent medium particularly at the visible wavelength region because of its extremely thin thickness. As for the Au NCs array, the maximum absorptivity reaches about 0.64 at 400 nm because LSPRs mode is activated. For a periodic Au NCs array on monolayer MoS_2_, the absorption is significantly enhanced compared with the monolayer MoS_2_. The maximum absorption peak appears at ~424 nm with the absorption of 0.65, while the other two relatively low peaks are at 620 and 665 nm, with absorption peaks of 0.51 and 0.45, respectively. The absorption of the structure is obviously improved, but it is still too weak for practical applications.

Taking this into consideration, the DBR nanostructure in this work was supported. The DBR nanostructure serves as a reflector to reabsorb the reflected light, and the influence on the photoelectric figure of the equipment can be neglected. The transmission of the nanostructure, which is the DBR combined with the Au NCs array, will increase, and the absorption of the monolayer MoS_2_ can be enhanced by the accomplishment of localized field concentration. It can be seen from the green line that the absorption is obviously improved due to the introduction of the DBR and the Au NCs array. The maximum absorption is above 0.99 from 533 to 554 nm, and another relatively low peak is above 0.94 at 422 nm.

Consequently, we investigated the influence of the period number *N* of DBR on the light absorption. With an increase in the period number of *N*, the absorption of nanostructures increases as shown in Figure 2b. When *N* = 1, the overall absorption is relatively low. The transmission of light is more obviously suppressed as the number *N* of DBR increases, thus improving light absorption. And the absorption will attain saturation when the period number *N* ≥ 5. The optical reflectivity of the inserted DBR, which limits its continued enhancement of absorption, achieves a saturated value. That is the reason why the period number *N* was set as 5 in our calculation, which can maximize the absorption and is relatively simple to achieve.

**Figure 3:**
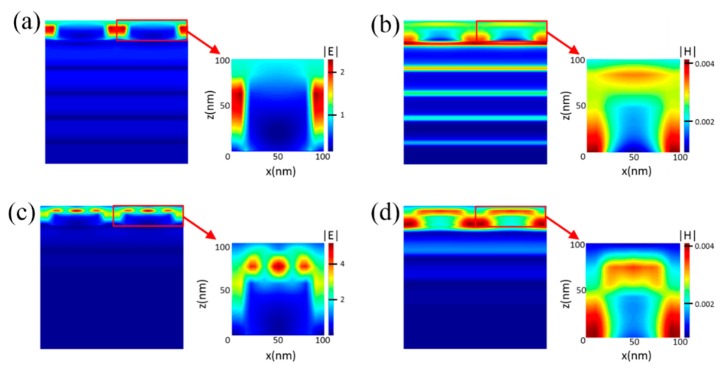
Electric field and magnetic field maps of the proposed MoS_2_-based nanostructure under TM-polarized light incidence. (**a**,**b**) represent the electric field and magnetic field maps at 422 nm. (**c**,**d**) represent the electric field and magnetic field maps at 545 nm.

Furthermore, the electric and magnetic influences on the two absorption peaks at the resonant wavelengths of 422 and 545 nm were investigated to explicate its physical mechanism. The distribution of electric field and magnetic field maps in a profile of the nanostructure under normal TM polarized light is shown in Figure 3a–d. As a result, the accumulation and the enhancement of electric and magnetic fields are clearly visible around the periphery of the Au nanocubes. In fact, the electromagnetic fields indicate such features due to the excitation of the LSPRs mode. MoS_2_ has a good second-order nonlinear susceptibility because the space-reversal symmetry is broken, and that is the physical foundation for obtaining a high-intensity second harmonic. It is known that the internal exciton resonance effect can enhance the second harmonic intensity [59,60], and the enhanced second harmonic intensity is closely related to the electric and magnetic field enhancement caused by the LSPRs. Therefore, the combination of monolayer MoS_2_ and an Au NCs array can enhance the absorption of monolayer MoS_2_ and then the overall absorption of the proposed nanostructure. To conclude, LSPRs results in the accumulation of light energy, near-field enhancement, and limiting the incident electric and magnetic fields near the surface of the monolayer MoS_2_ layer.

**Figure 4:**
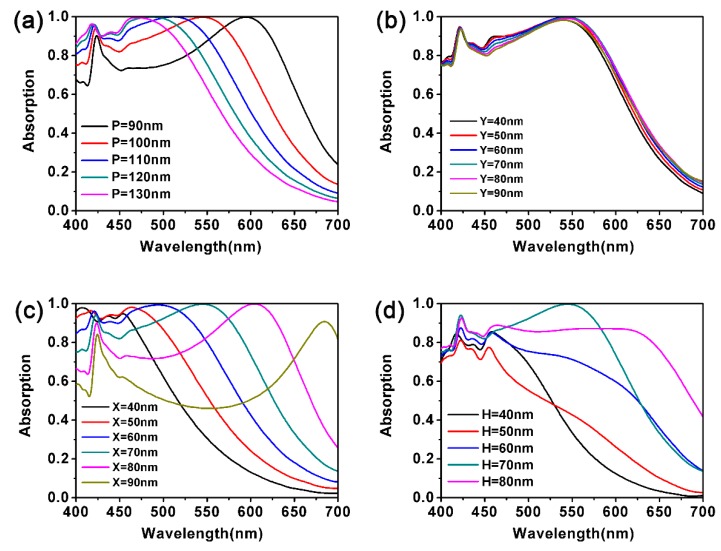
(**a**–**d**) Effects of different geometric parameters. The four pictures represent (**a**) the period of Au NCs array, (**b**) edge *Y* parallel to *Y*-axis, (**c**) edge *X* parallel to *X*-axis, and (**d**) the height *H* of the Au NCs.

Next, it was found that the LSPRs frequency of Au NPs can be effectively adjusted by geometric parameters and the surrounding medium [61,62,63], hence the period of the Au NCs array, the *X*, *Y,* and *H* of the cube, and the incident angles of light were investigated. For our cubic structure, the principle of influencing optical properties by changing its geometrical parameters is similar to that of gold nanorods [64]. Figure 4a shows the influence of the period of the Au NCs array on the light absorption rate of the MoS_2_-based nanostructure. When the period of the nanostructure increases from 90 to 130 nm, the absorption peak will appear at the shorter wavelength, which is due to the weakening of LSPRs between the Au NCs. With the increase of the period, the space between the nanocubes become larger, and the energy localized around them decreases, which means more energy is radiating out. Finally, these factors lead to a blue-shift. However, the overall peak does not change significantly, which provides possibilities for the operation of optoelectronic devices at different frequencies. Figure 4b demonstrates the effect of *Y* on the absorption spectra (here, we set *X* = *H* = 70 nm). It can be seen that there is no obvious change in the absorption spectra as a whole. This is because the magnetic field of TM-polarized light is perpendicular to the *x*–*z* plane and the change of the length of *Y* has little effect on absorption, which means that the resonant wavelength is not susceptible to *Y*. Subsequently, we then investigated the effects of *X* on the absorption spectra (*Y* and *H* are fixed as 70 nm). The results are shown in Figure 4c. Altering *X* from 40 to 90 nm, the effective resonant wavelength of LSPRs mode will increase, so the resonance peak would be obviously red-shifted from 454 to 685 nm. When *X* = 70 nm, the overall absorption rate achieves optimal value.

We then took the impacts of height (*H*) of the cubes on the absorptivity of monolayer MoS_2_ (*X* and *Y* are fixed as 70 nm) into account, as shown in Figure 4d. When the *H* is 70 nm, the peak value reaches a maximum of 0.99. Taking all the factors mentioned above into consideration, we finally decided to set *X* = *Y* = *Z* = 70 nm in this work.

**Figure 5:**
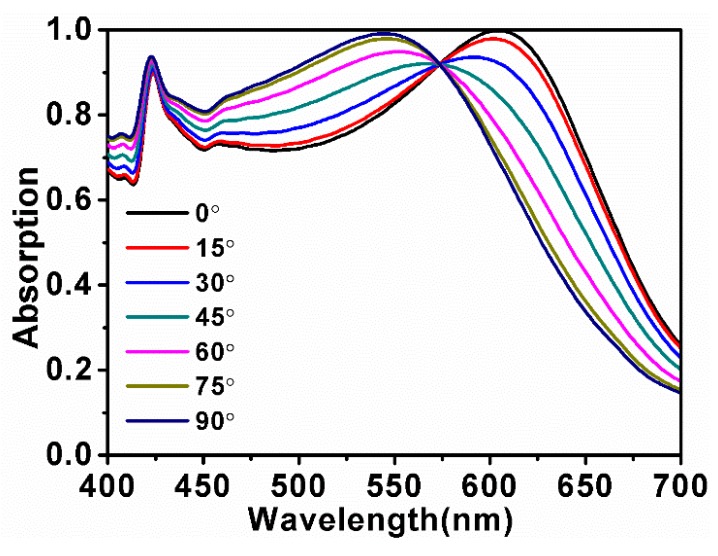
Absorption spectra with a different polarization direction (θ). *Y* and *H* are set as 70 nm and *X* is set as 80 nm. The θ increases from 0° to 90°.

**Figure 6:**
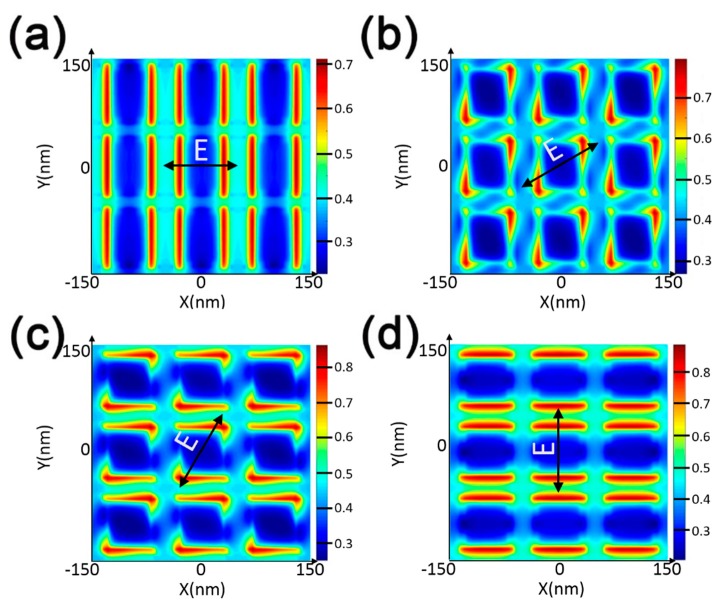
Electric field distributions (X–Y plane) on the surface for (**a**) θ = 0°, (**b**) θ = 30°, (**c**) θ = 60°, and (**d**) θ = 90°.

In addition, the effect of the polarization angle θ (θ is the angle between the plane wave and the *X*-axis) on structure absorption was discussed, as shown in Figure 5. It can be found that when the θ varies from 0° to 90°, the absorption peak blue-shifts from 607 to 544 nm. To understand the optical properties of the discussed polarization direction for the nanostructure, we simulated the electric field distributions (X–Y plane) on the surface (between the Au nanocubes and the monolayer MoS_2_) of the nanostructure, as illustrated in Figure 6. In Figure 6, the incident light is polarized along the *X*-axis (TM-polarized light). The θ increases from 0° to 90° and the electric field distributions of the cubes’ LSPRs excitation mode gradually changed from longitudinal dominance to transverse dominance, which is the main reason for the blue-shift of the absorption peak.

**Figure 7:**
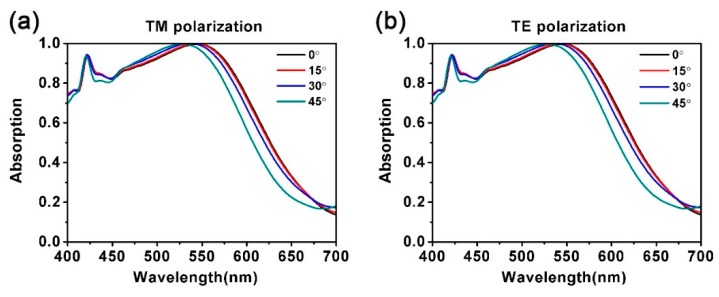
Influence of the incident angle on the absorption spectra for (**a**) TM polarization and (**b**) TE polarization.

Furthermore, we illustrated the absorption spectra at various incident angles for transverse electric (TE) and TM polarizations. As is widely known, in the pragmatic application of solar energy absorber, the ideal equipment should meet the requirements of high light absorption to adapt to a relatively large range of incident angles. The results are shown in Figure 7. When increasing the incident angle from 0° to 45°, the absorption peaks remained relatively stable, whether for TM or TE polarization, which means that both for TM or TE polarization, the absorption of monolayer MoS_2_ in the nanostructures can be significantly enhanced. This is because TM or TE polarized light with a small incident angle can keep LSPRs coupling. This provides a very suitable method for optimizing the absorption in the visible wavelength region by altering the incident angle. As a result, the combination of polarization insensitivity and excellent absorption stability at oblique incidence makes this kind of absorber more feasible in the pragmatic application of solar energy absorption [65,66,67,68].

**Figure 8:**
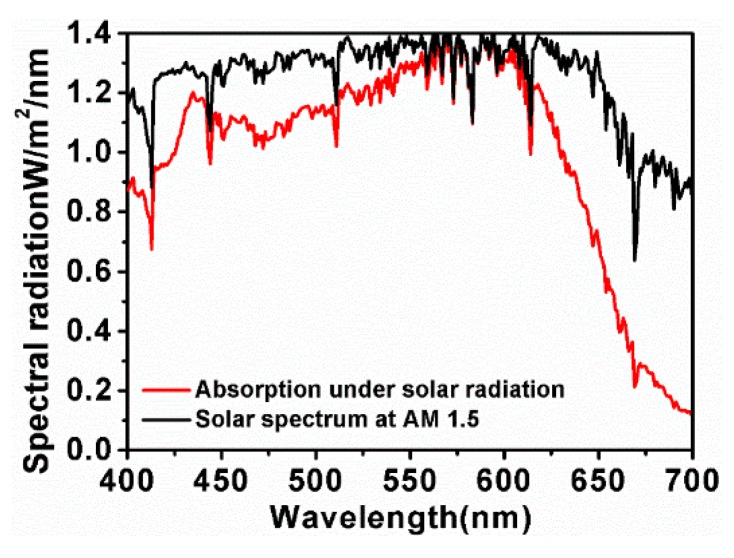
Absorption spectrum for the MoS_2_-based nanostructure absorber under a solar spectrum at AM 1.5.

In order to explore the value of the absorber in practical application, the solar energy absorption response of the proposed structure was studied. The results are shown in Figure 8. It can be observed that the proposed nanostructure exhibits a superior absorption performance for sunlight at the visible wavelength region under the solar source of Air Mass (AM) 1.5. As we can see from Figure 8, the average absorption can reach 90.5% at the wavelength range from 430 to 630 nm. Even in the whole region near the wavelength width of visible light, the average absorption is about 77.3%. Compared with several kinds of previous absorbers, there are many advantages in this work, as shown in Table 1. The average absorption rate of the proposed absorber is the highest and the range of the working area is also the widest, while the performance of other absorbers is somewhat inferior. The above researches confirm our assumption that the proposed nanostructure will absorb energy highly in the visible band of solar radiation.

**Figure 9:**
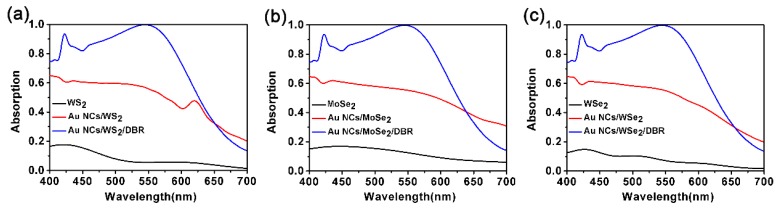
Light absorption rate of monolayer (**a**) WS_2_, (**b**) MoSe_2_ and (**c**) WSe_2_ in our nanostructures. The light absorption rate of Au NCs/TMDCs/DBR, Au NCs/TMDCs and monolayer TMDCs is shown in the figure as a comparison. *X* = *Y* = *H* = 70 nm, *P* = 100 nm, *d*_1_ = 38 nm, *d*_2_ = 90 nm, and *N* = 5. TMDCs: transition-metal dichalcogenides.

**Figure 10:**
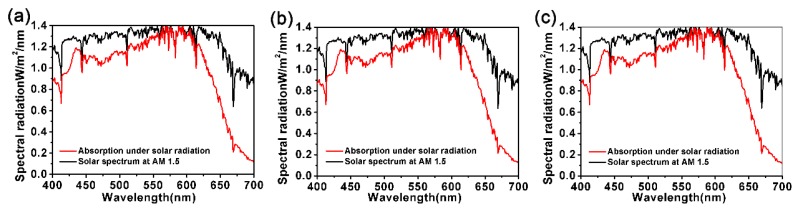
Absorption spectrums of monolayers (**a**) WS_2_, (**b**) MoSe_2_, and (**c**) WSe_2_ introduced into our nanostructure absorber under a solar spectrum at AM 1.5.

Finally, the proposed nanostructure was used to enhance the light absorption rate of other TMDCs, such as WS_2_, MoSe_2_, and WSe_2_, where MoS_2_ is displaced by other TMDCs in the proposed nanostructures. The light absorption rate for our proposed nanostructure of monolayer WS_2_, MoSe_2_ and WSe_2_ are shown in Figure 9a–c. The data for WS_2_, MoSe_2_ and WSe_2_ is obtained from Jiang et al. [37]. The selected geometric parameters are exactly the same as the proposed MoS_2_-based nanostructure. It can be seen that the absorption of these materials has been greatly enhanced by the nanostructure, too. The similarity between these three materials and MoS_2_ leads to this result, which also means that the operating wavelength can be changed by altering the geometric parameters mentioned above. It should be pointed out that due to the different corresponding imaginary parts of the dielectric constants of these three materials in close proximity to the resonance wavelength, different absorption losses are caused, resulting in slightly different absorption rates. Additionally, under the simulated solar irradiation, these three materials also show excellent solar energy absorption properties, as shown in Figure 10a–c. In conclusion, our proposed TMDCs-based nanostructure provides a theoretical basis for enhancing the solar energy absorption of other monolayer TMDCs materials.

## 4. Conclusions

A novel nanostructure composed of a periodic Au NCs array and distributed Bragg mirror (DBR) for a monolayer MoS_2_ was proposed to enhance LSPRs. By theoretical calculations, it was demonstrated that the combination of a monolayer MoS_2_ with an Au NCs array and a DBR provides a theoretical basis for the development of a perfect solar energy absorber with high-temperature stability, high absorptivity, and easy implementation. Under solar radiation, the absorption spectra generated by the absorber is well-matched with the energy distribution in the solar visible band. The light absorption rates of the proposed MoS_2_-based absorbers are more than 94.0% and 99.7% at the resonant wavelengths of 422 and 545 nm, respectively. Further studies show that the absorption of MoS_2_-based nanostructures can be flexibly adjusted by changing the geometric parameters and the polarization degrees of the incident light. This method has an important practical significance for improving the absorption efficiency and selectivity of monolayer MoS_2_. The proposed MoS_2_-based nanostructures can also be used for both TM and TE polarizations. Similar results can be obtained by other TMDCs, such as WS_2_, MoSe_2_, and WSe_2_. This will provide references for the design of other solar energy absorbers and optoelectronic devices based on TMDCs. The proposed structure will promote the areas of absorption efficiency, tunability and polarization-independence of solar energy absorbers once the large-scale fabrication techniques of such materials become more feasible in the future.

## Figures and Tables

**Table 1:** Comparison of several latest absorbers with the absorbers we have proposed. (quantum dots (QDs); metal-insulator-metal (MIM); graphene-molybdenum disulfide photovoltaic cells (GM-PVc); wedge-shaped metal-mirror microcavities (w-MMCs))

Absorber	Average Absorption (%)	Operating Region(nm)
Graphene/MoS_2_ Interface [69]	15.4%	Absorption is always less than 75%
QDs/MoS_2_/SiO_2_/Si [70]	22.3%	Absorption is always less than 75%
Monolayer MoS_2_ with MIM [44]	Less than 50%	500–750
GM-PVc in w-MMCs [71]	65%	450–700
Proposed (monolayer MoS_2_/Au NCs/DBR)	77.3%	400–700, absorbance is more than 90.4% from 430 to 630 nm
